# Concomitant systemic lupus erythematosus and HIV infection: A rare case report and literature review

**DOI:** 10.1097/MD.0000000000009337

**Published:** 2017-12-22

**Authors:** Hong-Yan Liao, Chuan-Min Tao, Jun Su

**Affiliations:** Department of Laboratory Medicine, West China Hospital, Sichuan University, Chengdu, Sichuan, China.

**Keywords:** acquired immunodeficiency syndrome, human immunodeficiency virus, lupus nephritis, systemic lupus erythematosus

## Abstract

**Rationale:**

Coexisting systemic lupus erythematosus (SLE) and human immunodeficiency virus (HIV) infection cases are rare worldwide. Great challenges are posed on the diagnosis and treatment of such concurrent cases.

**Patient concern:**

We report the case of a 44-year-old Chinese man with edema, hematuria, and fever who presented at West China Hospital, Sichuan University, Chengdu, Sichuan, China, in 2013.

**Diagnoses:**

An initial diagnosis of SLE was made from the clinical manifestations and laboratory findings based on the Systemic Lupus International Collaborating Clinics classification criteria. Immunosuppressant therapy relieved him of the edema and hematuria, but he regained the symptoms after a cold. Workup, including electrochemiluminescence immunoassay, western blot, and polymerase chain reaction analysis, revealed that he was concurrently infected with HIV after hospitalization.

**Interventions:**

The treatment plan included methylprednisolone and cyclophosphamide, with gastroprotective and hepatoprotective agents, simultaneously aiming to reduce urinary protein. After HIV infection confirmed, cyclophosphamide was stopped. He was referred to the local Centers for Disease Control and Prevention for combination antiretroviral therapy (ART). He was suggested to continue monitoring CD4 T-cell count for an appropriate dose of immunosuppressive drugs.

**Outcomes:**

In the last follow-up in May 2017, he had been stable in terms of both SLE and HIV infection.

**Lessons:**

The case highlights the presence of concurrent SLE and HIV infection. Laboratory technicians and clinicians should be cautious on diagnosis, especially in eliminating the false-positive results. Attention should be paid to the dose of immunosuppressants and the ART procedure.

## 1 Introduction

The coexisting cases of systemic lupus erythematosus (SLE) and human immunodeficiency virus (HIV) infection are rarely reported worldwide; however, studies on such cases may provide insight into the immunopathogenesis of the 2 disease conditions. Moreover, with caution, clinicians should evaluate the safety of SLE therapy based on immunosuppressants in the context of HIV infection. Therefore, interesting diagnostic and therapeutic dilemmas are raised in the case of coexistent SLE and HIV infection. We present a rare concomitant case of SLE and HIV infection, which has never been reported in China. We also provide a literature review and discuss the diagnostic, pathogenetic, and therapeutic implications of the association between these 2 diseases.

## 2 Materials and methods

### 2.1 Ethics statement

The use of the clinical data in this study has been approved by the Ethical Board of West China Hospital, Sichuan University.

### 2.2 HIV screening test

HIV screening procedure was performed using the Roche MODULAR ANALYTICS E170 immunoassay analyzer (Roche Diagnostics), as is described previously.^[1]^ Briefly, we used the Elecsys HIV Combi assay, a fourth-generation automated electrochemiluminescence immunoassay which is designed for the simultaneous detection of HIV p24 antigen, HIV-1, and HIV-2 antibodies. The analyzer automatically calculates the cutoff values based on the measurement of calibrations and the results are given in the form of a cutoff index (COI). Assay results are presented as ratios of specimen signals to the cutoff value (S/CO). Samples are considered as positive if COI or S/CO ≥1.0, as negative if the COI or S/CO <0.9 and as borderline if COI or S/CO is between 0.9 and 1.0.

### 2.3 HIV confirmatory tests

The HIV confirmatory tests include western blot HIV blot 2.2 (MP Diagnostics, Singapore) and COBAS AmpliPrep/COBAS TapMan HIV-1 Test (Roche Diagnostics), as is described previously.^[2]^ In China, HIV-1 western blots are usually interpreted following the National Guideline for Detection of HIV/AIDS (2009 edition), which require detection gp41 and gp120/160 (p24 and gp41/gp120/gp160) for positive results. The absence of all bands is a negative result. The result was recorded after reading by 2 different laboratory technologists and according to manufacturer's criteria for interpretation of positive results. Polymerase chain reaction (PCR) (COBAS AmpliPrep/COBAS TapMan HIV-1 Test, Roche Diagnostics) was used to quantitate HIV RNA levels and was conducted according to manufacturer's instructions. This was found to give a linear response from 48 HIV-1 RNA copies/mL to 10,000,000 HIV-1 RNA copies/mL and a sensitivity of ≤50 copies/mL across all subtypes ranging from <15 to 46 copies/mL of HIV-1 group M.

All the tests were fulfilled at Department of Medical Laboratory of West China Medical School/West China Hospital, Sichuan University, which has been certificated by COLLEGE OF AMERICAN PATHOLOGISTS (CAP) since 2006, and in 2008, 2010, 2012, 2014, and 2016 has passed the Laboratory accreditation review.

## 3 Case report

The patient was a 44-year-old Chinese man. In 2012, he started having headaches and dizziness for over 7 months, facial and edema in both lower limbs for 3 months, and gross hematuria for 1 month. He reported abdominal pain, fever, regurgitation, and belching, with occasional shortness of breath. He visited the local hospital. Complete blood count (CBC) demonstrated a hemoglobin (HGB) level of 64 g/L. Routine urinalysis revealed the following: protein (PRO; 2+), white blood cells (WBCs; 2+, 31/HP), and red blood cells (RBCs; 3+, 75/HP). His creatine level was 189 μmol/L. Elevated antinuclear antibody (ANA) level (1:320, homogeneous and cytoplasmic), +dsDNA level (1:320), decreased complement component 3 (C3) level (0.366 g/L), and C4 level (0.101 g/L) were confirmed. He was diagnosed as having SLE and SLE lupus nephritis (SLE-LN), and accepted prednisone (60 mg qd) and cyclophosphamide (50 mg bid) treatment in the local hospital. After the inpatient therapy, he was gradually relieved of the edema, headache, dizziness, and abdominal discomfort, but retained bubbling urine. In January 2013, he caught a cold. He regained eyelid and facial edemas after oral intake of penicillin. The edema gradually spread to both lower limbs and the whole body, accompanied by gross hematuria. He was admitted to the Department of Urology, West China Hospital.

Workup was conducted after hospitalization. Results of the investigations performed are shown in Table 1. CBC and differential demonstrated RBC counts 3.56 × 10^12^/L (4.3–5.8 × 10^12^/L), HGB level (102 g/L, 130–175 g/L), platelet count (16 × 10^9^/L, 100–300 × 10^9^), WBC count (6.36 × 10^9^/L, 3.5–9.5 × 10^9^), and neutrophil percentage (75.3%, 40%–75%). His platelet count decreased progressively as shown in the multiple CBC tests performed later. Routine urinalysis revealed the following results: PRO 2+ (−), WBC 1+, 9/HP (0–5/HP), RBC > +++, 3343/HP (0–3/HP). ANA +1:100 speckled (−), dsDNA – (−), C3 0.676 g/L (0.785–1.520 g/L), and C4 0.156 g/L (0.145–0.360 g/L) were also reported. Biochemical examination revealed the following values: alanine aminotransferase 42 IU/L (<50 IU/L), aspartate aminotransferase 19 IU/L (<50 IU/L), total PRO 62.4 g/L (65.0–85.0 g/L), albumin 39.7 g/L (40.0–55.0 g/L), creatine 199.7 μmol/L (53.0–140.0 μmol/L), glomerular filtration rate 46.98 mL/(min·1.73 m^2^) (56–122 mL/[min·1.73 m^2^]), cystatin C (CYC) 2.21 mg/L (0.51–1.09 mg/L), and 24-hour total urine PRO (24-TP) 1.57 g/24 h (<0.15 g/24 h). In accordance with the Systemic Lupus International Collaborating Clinics (SLICC) classification criteria,^[3]^ he was diagnosed of SLE, SLE-LN, and chronic kidney disease (stage 3). The treatment plan included methylprednisolone (40 mg ivgtt qd) and cyclophosphamide (50 mg ivgtt qd), with gastroprotective and hepatoprotective agents, simultaneously aiming to reduce urinary PRO level.

**Table 1:**
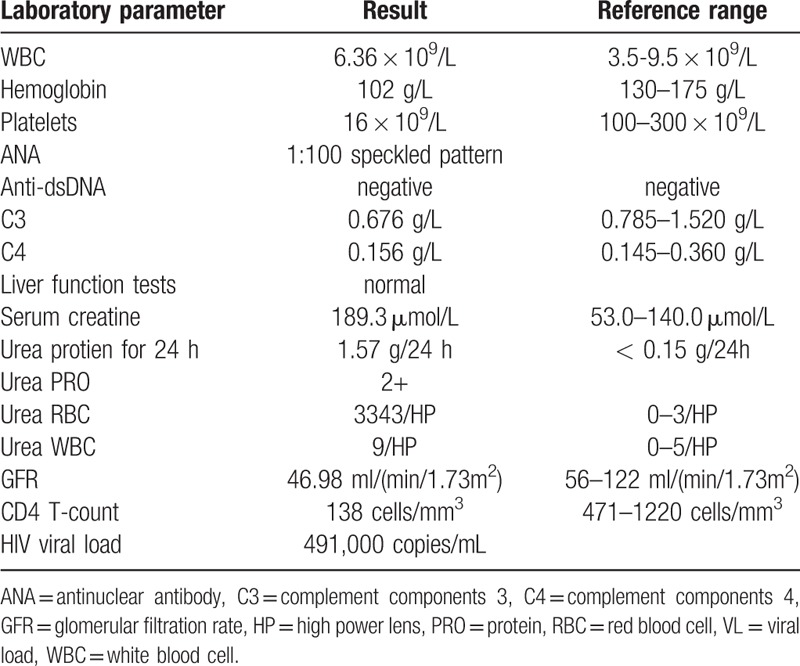
Results of laboratory investigations in West China Hospital.

A routine pretransfusion test revealed a highly suspected HIV-positive result as HIV electrochemiluminescence immunoassay result was 1,799,000 (cut-off value = 1) cut of index (COI) on January 28, 2013. The CD4 T-cell count was 138 cells/mm^3^, and the CD4^+^/CD8^+^ level was 0.48 (normal, 0.97–2.31). On January 31, 2013, western blot confirmed HIV infection with all bands positive. The HIV viral load (VL) was 491,000 copies/mL. He developed HIV infection with a known SLE history. Considering the low CD4 T-cell count, cyclophosphamide was stopped to reduce the chance of infection.

After the HIV infection was confirmed, cyclophosphamide was stopped. He was discharged and was suggested to transfer to the local Center for Disease Control and Prevention (CDC) for highly active antiretroviral therapy (HAART). The HAART included stavudine (d4T; 40 mg bid), lamivudine (3TC; 150 mg bid), and nevirapine (NVP; 200 mg bid) was initiated immediately. He underwent a routine workup that included CBC count, urinalysis routine, biochemical examination, ANA, C3 and C4 analyses, CD4 T-cell count, and HIV VL determination. He was kept on a regimen of oral prednisone (5 mg qd) to maintain a stable SLE condition. The latest test results in May 2017 showed the following: HGB 125 g/L, platelet 115 × 10^9^/L, serum creatine 111 μmol/L, urinary PRO 0.3 (+), RBC (−), WBC (−), ANA +1:100 (speckled), dsDNA (−), C3 0.771 g/L, and C4 0.150 g/L. His CD4 T-cell count was 460 cells/mm^3^ and CD4^+^/CD8^+^ level was 1.42. The HIV VL was <50 copies/mL. He was stable according to the follow-up.

### 3.1 Literature review

We identified 76 cases from all the English and Chinese literatures that reported concomitant HIV infection and SLE with sufficient detail from 1988 to the end of August 2017, using the keywords “systemic lupus erythematosus” and “AIDS” or “HIV” via PubMed and Medline. The current case is the first reported case of coexisting SLE and HIV infection in a Chinese patient. The following section describes the identified case reports subdivided chronologically depending on the timing of disease presentation or diagnosis.

Thirty-four patients had HIV infection followed by SLE. Of these individuals, 13 were pediatric patients between ages 7 months and 18 years.^[4–9]^ Seven from the pediatric group acquired HIV infection congenitally. One of the children presented initially with manifestations of SLE and then showed signs of congenital HIV infection.^[8]^ Positive lupus serologies, including anti-dsDNA antibodies, were found in these cases. Noteworthy, children with HIV infection and concurrent SLE usually develop manifestations of renal disease, such as focal glomerulosclerosis, mesangial hyperplasia, and LN.^[4,5]^ Such result is consistent with the recent findings that earlier onset of SLE may involve more renal features.^[10]^ However, the etiology of renal involvement in cases with coexisting SLE and HIV is difficult to determine. Renal complications due to HIV are also diverse.^[11]^ Without a definitive etiology, therapy in such patients is extremely difficult. Six deaths have been reported, all of which were in infected cases and cases of HIV diagnosed before the age of 10 years, except for 1 congenitally infected child without follow-up. The diseased children >10 years were all reported to remain stable or show improvement of HIV infection and SLE conditions under antiretroviral therapy (ART) and a lupus-based regimen (chloroquine [CQ], corticosteroids [CS], hydroxychloroquine [HCQ], and/or mycophenolate [MMF]). According to these results, it seems that earlier onset of SLE and HIV infection is fatal. Twenty-one adult patients developed SLE after HIV infection,^[6,12–26]^ with the age at HIV diagnosis ranging from 23 to 47 years (median, 38 years). Most adult patients (16/21, 80.0%) were females, in accordance with the reported frequency ratio of 4.3–9:1 (female-to-male ratio).^[27,28]^ The CD4 T-cell count at the time of SLE diagnosis was <500 cells/mm^3^ in 15 patients and >500 cells/mm^3^ in 2 patients, and was not reported in 4 patients. Clinical manifestations included respiratory distress, neuropathy, abnormal laboratory findings, and other associated symptoms. These patients were reported to respond well to treatment with CQ, and oral CS with or without HCQ. A simultaneous ART also helped them control the HIV infection, as all the patients reported improved or stable HIV infection. Hence, none of them were reported dead and most of them achieved partial, if not complete, remission.

Thirty-two patients died of SLE followed by HIV infection.^[6,16,29–50]^ The age of SLE diagnosis ranged from 18 to 55 years (median, 30 years), while most patients (26/32, 81.2%) were females, consistent with the known female predominance of SLE incidence. The duration of SLE before the diagnosis of HIV infection ranged from 2 weeks to 204 months (median duration, 72 months). Eighteen patients received ART, 10 did not, and 4 had no available information on ART. The patients were treated with SLE therapy, including CS, CQ, HCQ, CYC, and/or MMF, except for 2 patients who lacked the pertinent information, and most of them exhibited inactive SLE after the onset of HIV infection regardless of the use of ART. One patient developed a lupus flare 7 months after ART^[30]^. One patient developed LN 13 months after ART.^[50]^ Two patients progressed to end-stage renal disease.^[6,48]^ Seven died of acquired immunodeficiency syndrome (AIDS)-related infection, despite 3 of them receiving ART. Two of them were diagnosed as having HIV infection after death.

We also identified 10 cases featured by a simultaneous diagnosis of SLE and HIV during the same admission.^[14,16,51–53]^ However, the precise timing of onset of either condition is difficult to ascertain for some patients considering the long duration between seroconversion and diagnosis of HIV infection in some patients.^[54]^ The age at diagnosis ranged from 18 to 44 years (median, 26 years). Again, female was the predominant sex in this group (8/10, 80%). Three patients were not receiving ART before and after diagnosis, among which 1 died of meningitis infection^[53]^ and the other 2 developed lupus flare after months.^[14]^ The rest of the patients tolerated ART and exhibited inactive or improved SLE and HIV, except that 1 patient who initially had inactive SLE developed flare after receiving ART for 82 months.^[14]^

## 4 Discussion and conclusion

Zhang et al reported a seminal study on the spectrum and characteristics of rheumatic complications in HIV patients in China and described 1 HIV-infected patient presenting with lupus-like syndrome.^[55]^ However, it is unknown whether this fulfills the American College of Rheumatology or the SLICC criteria for SLE diagnosis. Herein, we present the first concurrent case of SLE and HIV infection in China. The diagnosis is achieved by combination of the clinical presentation, laboratory findings, and medical history. Interestingly, the diagnosis was more coincidental than a result of stringent reasoning. The accurate times of the onsets of SLE and HIV infection were also unknown. He was referred to our hospital again because he caught a cold and regained the symptoms. Therefore, he was likely to be infected with HIV infection after hospital discharge, that is, he developed HIV infection with a SLE history. During hospitalization, a renal biopsy was not executed considering the low platelet count, which may have been resulted from HIV-associated immune thrombocytopenic purpura or the side effect of cyclophosphamide. As long as the coexistence is confirmed, therapeutic intervention is required, considering that a immunosuppressant dose is crucial to the pathogenesis of AIDS disease progression. This patient was referred to the CDC to control the HIV infection via ART first and keeps a routine monitoring of CD4 T-cell count and HIV VL. Accordingly, he was given SLE therapy. Under such management, he remained stable in terms of both HIV infection and SLE until the recent follow-up.

Since the first case reported in 1988,^[31]^ only a few cases of concurrent SLE and HIV infection have been reported. Sporadic reported concurrent cases have attracted increasing attention to the potential association of SLE and HIV infection. In fact, these 2 conditions share multiple overlapping clinical features, including hematological, neurological, renal, and other abnormalities. Presentations with lymphopenia, hemolytic anemia, or thrombocytopenia are common in both disease sets. Both may display manifestations that include psychosis, peripheral neuropathy, and focal deficits. Nephropathy is another common manifestation of both diseases.^[6,56]^ Nonspecific symptoms of such wide range make great challenges to the diagnosis of concurrent cases and differential diagnosis of cases with mimicking clinical manifestations.

Overproduction of ANAs and anti-dsDNA antibodies is a hallmark of SLE. ANAs are of diagnostic, pathological, and prognostic significance during lupus disease course. Noteworthy, recent studies revealed polyreactivity or autoreactivity one of the key features of anti-HIV antibodies, especially antibodies with broad and potent neutralizing abilities.^[57–59]^ Although existing in low titer, these ANAs resulted from disrupted immunological milieu may play an etiological role in autoimmune diseases. Furthermore, patients with SLE may also produce antibodies cross-reacting with HIV antigens, therefore leading to false positive tests for HIV infection.^[60–64]^ HIV-infected individuals may also produce autoantibodies, such as ANAs and anticardiolipin antibodies,^[58,65]^ causing diagnostic difficulties.

Although the concomitant cases of SLE and HIV infection are limited, the isolated coexisting cases indicate that these 2 conditions are not mutually exclusive. As HIV is gradually affecting more heterosexual populations than homosexual men and female predominates in populations with lupus, more cases may be reported in the future. Therefore, elucidation of the underlying disease mechanisms and timely diagnosis should provide a better understanding of the pathogenesis, new therapeutic strategies, and improved care of patients. Laboratory technicians and clinicians should combine the results of western blot, PCR analysis, and CD4 T-cell count to exclude false-positive result for HIV infection concurrent with SLE onset. For SLE patients with confirmed HIV infection, anti-HIV therapy should be considered before immunosuppressive treatment. Moreover, monitoring of CD4 T-cell count is strongly recommended in terms of determining the appropriate immunosuppressant dose.

## References

[1] Wang T Li D Yan K Performance evaluation of a new fourth-generation HIV Ag/Ab combination electrochemiluminescence immunoassay—evaluation of a new HIV assay. Int J STD AIDS 2014;25:267–72. 23970655 10.1177/0956462413499909

[2] Bi X Ning H Wang T Comparative performance of electrochemiluminescence immunoassay and EIA for HIV screening in a multiethnic region of China. PLoS One 2012;7:e48162. 23144740 10.1371/journal.pone.0048162 PMC3483174

[3] Petri M Orbai AM Alarcon GS Derivation and validation of the Systemic Lupus International Collaborating Clinics classification criteria for systemic lupus erythematosus. Arthritis Rheum 2012;64:2677–86. 22553077 10.1002/art.34473 PMC3409311

[4] D’Agati V Seigle R Coexistence of AIDS and lupus nephritis: a case report. Am J Nephrol 1990;10:243–7. 2382686 10.1159/000168090

[5] Strauss J Abitbol C Zilleruelo G Renal disease in children with the acquired immunodeficiency syndrome. N Engl J Med 1989;321:625–30. 2770791 10.1056/NEJM198909073211001

[6] Chang BG Markowitz GS Seshan SV Renal manifestations of concurrent systemic lupus erythematosus and HIV infection. Am J Kidney Dis 1999;33:441–9. 10070907 10.1016/s0272-6386(99)70180-0

[7] O’Keefe K Edelheit B Onel K Systemic lupus erythematosus in a pediatric patient with congenital acquired immunodeficiency syndrome. Pediatr Infect Dis J 2001;20:450–2. 11332676 10.1097/00006454-200104000-00018

[8] Chalom EC Rezaee F Mendelson J Pediatric patient with systemic lupus erythematosus & congenital acquired immunodeficiency syndrome: An unusual case and a review of the literature. Pediatr Rheumatol Online J 2008;6:7. 18452619 10.1186/1546-0096-6-7 PMC2390549

[9] Mialou V Bertrand Y Bouvier R Lupus nephritis in a child with AIDS. Am J Kidney Dis 2001;37:E27. 11273897 10.1016/s0272-6386(01)90013-7

[10] Aljohani R Gladman DD Su J Disease evolution in late-onset and early-onset systemic lupus erythematosus. Lupus 2017;26:1190–6. 28420066 10.1177/0961203317696593

[11] Nebuloni M Barbiano di Belgiojoso G Genderini A Glomerular lesions in HIV-positive patients: a 20-year biopsy experience from Northern Italy. Clin Nephrol 2009;72:38–45. 19640386 10.5414/cnp72038

[12] Virot E Duclos A Adelaide L Autoimmune diseases and HIV infection: a cross-sectional study. Medicine (Baltimore) 2017;96:e5769. 28121924 10.1097/MD.0000000000005769 PMC5287948

[13] de Oliveira LR Ferreira TC Neves Fde F Aplastic anemia associated to systemic lupus erythematosus in an AIDS patient: a case report. Rev Bras Hematol Hemoter 2013;35:366–8. 24255622 10.5581/1516-8484.20130100 PMC3832319

[14] Mody GM Patel N Budhoo A Concomitant systemic lupus erythematosus and HIV: case series and literature review. Semin Arthritis Rheum 2014;44:186–94. 24913962 10.1016/j.semarthrit.2014.05.009

[15] Daikh BE Holyst MM Lupus-specific autoantibodies in concomitant human immunodeficiency virus and systemic lupus erythematosus: case report and literature review. SeminArthritis Rheum 2001;30:418–25. 10.1053/sarh.2001.23149 11404825

[16] Furie RA Effects of human immunodeficiency virus infection on the expression of rheumatic illness. Rheum Dis Clin North Am 1991;17:177–88. 2041886

[17] Maradona JA Carton JA Asensi V Myasthenia gravis and systemic lupus erythematosus in association with human immunodeficiency virus infection. Clin Infect Dis 1995;20:1577–8. 7548525 10.1093/clinids/20.6.1577

[18] Contreras G Green DF Pardo V Systemic lupus erythematosus in two adults with human immunodeficiency virus infection. Am J Kidney Dis 1996;28:292–5. 8768929 10.1016/s0272-6386(96)90317-0

[19] Kudva YC Peterson LS Holley KE SLE nephropathy in a patient with HIV infection: case report and review of the literature. J Rheumatol 1996;23:1811–5. 8895165

[20] Smith PR D’Cruz D Raftery MJ Drug-induced lupus nephritis in HIV infection. Rheumatology (Oxford) 1999;38:1017–8. 10534557 10.1093/rheumatology/38.10.1017

[21] Alonso CM Lozada CJ Effects of IV cyclophosphamide on HIV viral replication in a patient with systemic lupus erythematosus. Clin Exp Rheumatol 2000;18:510–2. 10949730

[22] Calza L Manfredi R Colangeli V Systemic and discoid lupus erythematosus in HIV-infected patients treated with highly active antiretroviral therapy. Int J STD AIDS 2003;14:356–9. 12803945 10.1258/095646203321605585

[23] Asherson RA Gomez-Puerta JA Marinopoulos G Recurrent pulmonary thromboembolism in a patient with systemic lupus erythematosus and HIV-1 infection associated with the presence of antibodies to prothrombin: a case report. Clin Infect Dis 2005;41:e89–92. 16231247 10.1086/497369

[24] Schneider J Zatarain E IRIS and SLE. Clin Immunol 2006;118:152–3. 16376154 10.1016/j.clim.2005.09.004

[25] Jakez-Ocampo J Carrillo-Maravilla E Richaud-Patin Y An unusual multiplex systemic lupus erythematosus family with high prevalence of nephropathy, late-onset disease, and one member with disease-onset post-HIV therapy. J Clin Rheumatol 2008;14:34–7. 18431097 10.1097/RHU.0b013e3181639abe

[26] Abbott IJ Chang CC Skinner MJ Development and management of systemic lupus erythematosus in an HIV-infected man with hepatitis C and B co-infection following interferon therapy: a case report. J Med Case Rep 2009;3:7289. 19830165 10.4076/1752-1947-3-7289 PMC2726516

[27] Crowson CS Matteson EL Myasoedova E The lifetime risk of adult-onset rheumatoid arthritis and other inflammatory autoimmune rheumatic diseases. Arthritis Rheum 2011;63:633–9. 21360492 10.1002/art.30155 PMC3078757

[28] Gleicher N Barad DH Gender as risk factor for autoimmune diseases. J Autoimmun 2007;28:1–6. 17261360 10.1016/j.jaut.2006.12.004

[29] Hazarika I Chakravarty BP Dutta S Emergence of manifestations of HIV infection in a case of systemic lupus erythematosus following treatment with IV cyclophosphamide. Clin Rheumatol 2006;25:98–100. 16132163 10.1007/s10067-005-1133-6

[30] Drake WP Byrd VM Olsen NJ Reactivation of systemic lupus erythematosus after initiation of highly active antiretroviral therapy for acquired immunodeficiency syndrome. J Clin Rheumatol 2003;9:176–80. 17041454 10.1097/01.RHU.0000073591.34503.4e

[31] Kopelman RG Zolla-Pazner S Association of human immunodeficiency virus infection and autoimmune phenomena. Am J Med 1988;84:82–8. 3257353 10.1016/0002-9343(88)90012-5

[32] Fox RA Isenberg DA Human immunodeficiency virus infection in systemic lupus erythematosus. Arthritis Rheum 1997;40:1168–72. 9182929 10.1002/art.1780400623

[33] Yeh CK Fox PC Goto Y Human immunodeficiency virus (HIV) and HIV infected cells in saliva and salivary glands of a patient with systemic lupus erythematosus. J Rheumatol 1992;19:1810–2. 1491408

[34] Bambery P Deodhar SD Malhotra HS Blood transfusion related HBV and HIV infection in a patient with SLE. Lupus 1993;2:203–5. 8369814 10.1177/096120339300200315

[35] Jindal R Solomon M Burrows L False positive tests for HIV in a woman with lupus and renal failure. N Engl J Med 1993;328:1281–2. 8464453 10.1056/NEJM199304293281717

[36] Povolotsky J Polsky B Laurence J Withdrawal of conclusion: false positive tests for HIV in a woman with lupus. N Engl J Med 1994;331:881–2. 10.1056/NEJM199409293311318 8078545

[37] Itoh K Nishioka Y Hirohata S HIV induced systemic lupus erythematosus. Lupus 1994;3:205–6. 7951307 10.1177/096120339400300313

[38] Lu I Cohen PR Grossman ME Multiple dermatofibromas in a woman with HIV infection and systemic lupus erythematosus. J Am Acad Dermatol 1995;32 (5 pt 2):901–3. 7722054 10.1016/0190-9622(95)91558-3

[39] Molina JF Citera G Rosler D Coexistence of human immunodeficiency virus infection and systemic lupus erythematosus. J Rheumatol 1995;22:347–50. 7738963

[40] Byrd VM Sergent JS Suppression of systemic lupus erythematosus by the human immunodeficiency virus. J Rheumatol 1996;23:1295–6. 8823711

[41] Cimmino MA De Maria A Moggiana G HIV infection in a male patient with systemic lupus erythematosus. Clin Exp Rheumatol 1996;14:317–20. 8809449

[42] Fernandez-Miranda C Rubio R Pulido F Development of acquired immune deficiency syndrome in a patient with systemic lupus erythematosus. J Rheumatol 1996;23:1308. 8823717

[43] Learmont JC Geczy AF Mills J Immunologic and virologic status after 14 to 18 years of infection with an attenuated strain of HIV-1. A report from the Sydney Blood Bank Cohort. N Engl J Med 1999;340:1715–22. 10352163 10.1056/NEJM199906033402203

[44] Clutterbuck DJ Watson J De Ruiter A Presence of HIV infection in patients diagnosed with systemic lupus erythematosus. Rheumatology (Oxford) 2000;39:1047–8. 10.1093/rheumatology/39.9.1047-a 10986318

[45] Wanchu A Sud A Singh S Human immunodeficiency virus infection in a patient with systemic lupus erythematosus. J Assoc Physicians India 2003;51:1102–4. 15260397

[46] Colon M Martinez DE Clinical remission of systemic lupus erythematosus after human immunodeficiency virus infection. P R Health Sci J 2007;26:79–81. 17674878

[47] Kaliyadan F Hiv and lupus erythematosus: a diagnostic dilemma. Indian J Dermatol 2008;53:80–2. 19881993 10.4103/0019-5154.41652 PMC2763717

[48] Yao Q Frank M Glynn M Rheumatic manifestations in HIV-1 infected in-patients and literature review. Clin Exp Rheumatol 2008;26:799–806. 19032811

[49] Burton J Vera JH Kapembwa M HIV and systemic lupus erythematosus: the clinical and diagnostic dilemmas of having dual diagnosis. Int J STD AIDS 2010;21:845–6. 21297099 10.1258/ijsa.2010.010062

[50] Habibi S Agrawal S Narsimulu G Mycophenolate: dual utility in rare conditions of HIV infection complicating lupus. Lupus 2010;19:774–5. 20156930 10.1177/0961203309355809

[51] Palacios R Santos J Valdivielso P Human immunodeficiency virus infection and systemic lupus erythematosus. An unusual case and a review of the literature. Lupus 2002;11:60–3. 11898923 10.1191/0961203302lu141cr

[52] Carugati M Franzetti M Torre A Systemic lupus erythematosus and HIV infection: a whimsical relationship. Reports of two cases and review of the literature. Clin Rheumatol 2013;32:1399–405. 23649483 10.1007/s10067-013-2271-x

[53] Gould T Tikly M Systemic lupus erythematosus in a patient with human immunodeficiency virus infection–challenges in diagnosis and management. Clin Rheumatol 2004;23:166–9. 15045634 10.1007/s10067-003-0833-z

[54] Fox C Walker-Bone K Evolving spectrum of HIV-associated rheumatic syndromes. Best Pract Res Clin Rheumatol 2015;29:244–58. 26362742 10.1016/j.berh.2015.04.019 PMC4759923

[55] Zhang X Li H Li T Distinctive rheumatic manifestations in 98 patients with human immunodeficiency virus infection in China. J Rheumatol 2007;34:1760–4. 17659750

[56] Iordache L Launay O Bouchaud O Autoimmune diseases in HIV-infected patients: 52 cases and literature review. Autoimmun Rev 2014;13:850–7. 24747058 10.1016/j.autrev.2014.04.005

[57] Liao HX Chen X Munshaw S Initial antibodies binding to HIV-1 gp41 in acutely infected subjects are polyreactive and highly mutated. J Exp Med 2011;208:2237–49. 21987658 10.1084/jem.20110363 PMC3201211

[58] Liu M Yang G Wiehe K Polyreactivity and autoreactivity among HIV-1 antibodies. J Virol 2015;89:784–98. 25355869 10.1128/JVI.02378-14 PMC4301171

[59] Mouquet H Scheid JF Zoller MJ Polyreactivity increases the apparent affinity of anti-HIV antibodies by heteroligation. Nature 2010;467:591–5. 20882016 10.1038/nature09385 PMC3699875

[60] Esteva MH Blasini AM Ogly D False positive results for antibody to HIV in two men with systemic lupus erythematosus. Ann Rheum Dis 1992;51:1071–3. 1417140 10.1136/ard.51.9.1071 PMC1004841

[61] Ranki A Kurki P Riepponen S Antibodies to retroviral proteins in autoimmune connective tissue disease. Relation to clinical manifestations and ribonucleoprotein autoantibodies. Arthritis Rheum 1992;35:1483–91. 1472125 10.1002/art.1780351212

[62] Barthel HR Wallace DJ False-positive human immunodeficiency virus testing in patients with lupus erythematosus. Semin Arthritis Rheum 1993;23:1–7. 8235661 10.1016/s0049-0172(05)80021-6

[63] Coll J Palazon J Yazbeck H Antibodies to human immunodeficiency virus (HIV-1) in autoimmune diseases: primary Sjogren's syndrome, systemic lupus erythematosus, rheumatoid arthritis and autoimmune thyroid diseases. Clin Rheumatol 1995;14:451–7. 7586984 10.1007/BF02207681

[64] Scherl M Posch U Obermoser G Targeting human immunodeficiency virus type 1 with antibodies derived from patients with connective tissue disease. Lupus 2006;15:865–72. 17211992 10.1177/0961203306071405

[65] Massabki PS Accetturi C Nishie IA Clinical implications of autoantibodies in HIV infection. AIDS 1997;11:1845–50. 9412703 10.1097/00002030-199715000-00009

